# Effectiveness of Life Goal Framing to Motivate Medical Students During Online Learning: A Randomized Controlled Trial

**DOI:** 10.5334/pme.1017

**Published:** 2023-10-26

**Authors:** Adam G. Gavarkovs, Jeff Crukley, Erin Miller, Rashmi A. Kusurkar, Kulamakan Kulasegaram, Ryan Brydges

**Affiliations:** 1 Institute of Health Policy, Management and Evaluation, University of Toronto, Toronto, Ontario, Canada; 2 The Wilson Centre, University Health Network, Toronto, Ontario, Canada; 3 Data Science and Statistics, Toronto, Ontario, Canada; 4 Department of Speech-Language Pathology, Temerty Faculty of Medicine, University of Toronto, Toronto, Ontario, Canada; 5 Department of Psychology, Neuroscience, and Behavior, McMaster University, Hamilton, Ontario, Canada; 6 School of Physical Therapy, Faculty of Health Sciences, Western University, London, Ontario, Canada; 7 Amsterdam UMC location Vrije Universiteit, Amsterdam, De Boelelaan 1118, The Netherlands; 8 LEARN! Research Institute for Learning and Education, Faculty of Psychology and Education, VU University Amsterdam, Amsterdam, The Netherlands; 9 Amsterdam Public Health, Quality of Care, Amsterdam, The Netherlands; 10 Department of Family & Community Medicine, and Temerty Chair in Learner Assessment and Program Evaluation, Temerty Faculty of Medicine, University of Toronto, Toronto, Ontario, Canada; 11 Department of Medicine, Temerty Faculty of Medicine, University of Toronto, Toronto, Ontario, Canada; 12 Professorship in Technology-Enabled Education at St. Michael’s Hospital, Unity Health Toronto, Toronto, Ontario, Canada

## Abstract

**Introduction:**

Educators need design strategies to support medical students’ motivation in online environments. Prompting students to frame a learning activity as preparing them to attain their life goals (e.g., helping others) via their clinical practice, a strategy called ‘life goal framing’, may enhance their autonomous motivation, learning strategy use, and knowledge retention. However, for students with low perceived competence for learning (PCL), life goal framing may have an adverse effect. A randomized controlled trial was conducted to test the effectiveness of life goal framing and the moderating effect of students’ PCL.

**Methods:**

First- and second-year medical students across four Canadian universities (*n* = 128) were randomized to receive a version of an online module with an embedded prompt for life goal framing, or one without. Students’ motivation, learning strategy use, and knowledge retention were assessed. Differences between conditions on each outcome were estimated using Bayesian regression.

**Results:**

Students’ PCL was a moderator for autonomous motivation but no other outcomes. The prompt did not have a statistically significant effect on any outcome, even for learners with high PCL, except for a small effect on link-clicking behaviour.

**Discussion:**

The results of this study suggest that learners’ autonomous motivation is influenced by how they make meaning of instruction in terms of their future life goals *and* their present confidence. We cannot recommend life goal framing as an effective design strategy at this point, but we point to future work to increase the benefit of life goal framing for learners with high confidence.

## Introduction

Post-pandemic health professions education (HPE) continues to rely heavily on the internet. Online instruction can help educators respond to perennial challenges such as reaching geographically distributed learners [1] and adapting to learners’ busy schedules [2]. However, certain instructional design strategies can make online instruction more or less effective [3,4,5]. Accordingly, researchers must identify effective design strategies to provide educators with the tools to design more effective online instruction [6,7]. A particularly vexing issue for educators involves designing *motivating* online instruction [5,8].

Students with better motivation tend to report using more deep learning strategies, spend more time on learning tasks, and demonstrate greater achievement, including in online learning contexts [9,10,11,12,13]. Though certain instructional design strategies can influence students’ motivation, research on effective motivational design strategies in HPE is sparse [14,15,16]. Without evidence-based guidance, educators may be producing online instruction that sub-optimally supports, or even thwarts, students’ motivation to learn.

The aim of this study is to investigate a novel motivational design strategy we call ‘prompting life goal framing’ [17]. Life goal framing interventions aim to make some reasons for engaging with instruction more salient than others, toward enhancing learners’ their motivation, self-regulation, and achievement. We begin by presenting a theoretical account of motivated engagement in instructional activities [17], building upon principles from Control Theory [18] and Self-Determination Theory (SDT). We then use this theoretical account to generate predictions regarding the effects of a life goal framing prompt and present a randomized controlled trial (RCT) testing these predictions.

### A theoretical account of motivated engagement in instructional activities

Control Theory construes engagement with instruction as goal-directed and controlled by a feedback loop [18]. When learners sit down to complete instruction (e.g., an e-learning module), they set goals for what they want to accomplish, and then use learning strategies that they think will help them attain their goals. Learners are often documented as using three different types of strategies to help them learn: *rehearsal strategies* (e.g., highlighting, taking verbatim notes), which facilitate selecting new information into working memory, *organization strategies* (e.g., creating lists or concept maps), which facilitate constructing relations among new pieces of information, and *elaboration strategies* (e.g., self-explaining), which facilitate integrating new information with prior knowledge [19]. Compared to rehearsal strategies, organization and elaboration strategies appear to afford a deeper understanding of new information [19,20]. As learners persist toward their goals, they may check their progress through *metacognitive monitoring*, which involves attending to information that signals something about their current understanding (e.g., answers to self-test questions), and then comparing this current state to their goal state [21]. Learners can adapt how they approach an activity in response to metacognitive monitoring [21].

SDT proposes that learners’ goal pursuit can be energized by different *types* of motivation, based on their reasons for wanting to attain their goals. Learners experience autonomous motivation when pursuing their goals stems from personal meaning or interest, whereas they experience controlled motivation when pursuing their goals stems from satisfying external demands (e.g., assessments) or quelling internal pressures (e.g., feelings of guilt or shame). Research in HPE has consistently demonstrated positive associations between autonomous motivation, study effort, and learners’ use of ‘deep’ strategies [9,10,11,22,23,24].

Taken together, Control Theory and SDT suggest that, when learners experience high levels of autonomous motivation, they will tend to engage more deeply in an activity (e.g., by using organization and elaboration strategies), monitor their progress regularly (e.g., by using metacognitive monitoring strategies), and demonstrate enhanced knowledge retention.

### Life goal framing: A proposed design strategy to enhance autonomous motivation

Learners often pursue a career in healthcare because it represents a *pathway* to become a desired kind of person or to make a desired impact on the world [25]. We refer to these overarching aims as *life goals*. When learners pursue their life goals, they work to manifest their ideal self [18]. Accordingly, learners’ life goals are among the most self-defining and meaningful goals that they possess [18].

We propose that learners can be prompted to frame an instructional activity as preparing them to attain their life goals via their future clinical practice. We refer to this motivational design strategy as ‘prompting life goal framing’. We predict that a life goal framing prompt may enhance the personal meaning that learners ascribe to instruction, thereby increasing their autonomous motivation to deeply understand the target concepts and skills [17,26]. The proposed effect on learners’ autonomous motivation may then, in turn, encourage greater use of organization, elaboration, and metacognitive strategies, and enhance knowledge retention.

A life goal framing prompt may not benefit all learners, however. As learners begin an activity, they can make expectancy judgements about whether they will attain their goals [27]. Learners with low *perceived competence for learning* (PCL), which refers to their confidence in their ability to achieve their learning goals, may form a negative expectancy judgement. For learners who connect their work on an activity to their life goals, a negative expectancy judgement may be highly threatening, because failure may feel threatening to life goal attainment [28]. Accordingly, we predict that if learners form negative expectancy judgements after connecting an activity to their life goals, they may experience *poorer* motivation than if they did not engage in life goal framing at all. Some [29,30], but not all [31], prior studies have found that the motivational effects of similar prompt-based strategies are moderated by learners’ PCL.

To test these predictions, we conducted an RCT among first- and second-year medical students, guided by two research questions:

**Research Question #1:** Do students who receive a prompt to engage in life goal framing within an online module demonstrate higher autonomous motivation, learning strategy use, and knowledge retention, and lower controlled motivation, compared to medical students who do not receive a prompt? Answering this question may provide educators with a motivational design strategy they can use to enhance learners’ motivation, self-regulation, and achievement when learning online.

**Research Question #2:** Does the effect of receiving a life goal framing prompt within an online module on students’ motivation, learning strategy use, and knowledge retention depend on their PCL? Answering this question will provide researchers and educators in HPE with a deeper understanding of the process by which learners make meaning of instructional activities and how this process influences their engagement.

## Method

### Sample

Our sample included first- and second-year students enrolled at four Canadian medical schools (total population = ~1,600 students). We aimed to recruit at least 90 participants (45 per arm) based on previous studies investigating instructional design strategies [3,29], informed by Norman and colleagues’ suggested approach [32]. The study was approved by the University of Toronto Office of Research Ethics Board.

### Study setting

We developed a bespoke module on the physiology of weight loss in collaboration with two subject matter experts. Three features of the module are worth noting. First, the module included 29 embedded hyperlinks to references and key resources. Second, the module included four ‘interactive’ sections where students could manipulate input variables (e.g., the macronutrient composition of a diet) and observe their effects (e.g., on the thermic effect of food). Third, the module included five ‘question’ slides where students were presented with a question (e.g., what strategies might help to attenuate the reduction in the thermic effect of food associated with weight loss?) and could click to reveal the answer. The module was designed to take about 45 minutes to complete. Four second- through fourth-year medical students piloted the module and reported that it reflected the right level of challenge for first- and second-year students. We made no subsequent changes to the module and pilot students did not participate in the trial.

### Intervention

The life goal framing prompt consisted of a single slide in the module’s introductory section, between the general introduction and learning objectives slides (see full text in Supplemental Digital Appendix 1). Informed by Control Theory and SDT, we designed the slide to help students connect what they were learning to the life goals they pursue through their career in medicine [17]. The prompt encouraged students to consider: (1) the clinical skills enabled by learning the module content (e.g., developing realistic and sustainable weight management plans for patients), and (2) how developing these skills might serve their life goals (e.g., making a meaningful difference in the lives of their patients). The prompt included a patient narrative from a member of Obesity Canada’s Public Engagement Committee, alongside their photograph. Students were asked to type a brief reflection in an embedded text box. We aimed to use noncontrolling language to support students’ autonomy [33]. A third-year medical student reviewed, edited, and approved the prompt text. The control group’s module was equivalent to that of the intervention group, save for this embedded activity.

### Procedure

After consenting to participate, students were randomized in blocks of six to the prompt or no-prompt condition to ensure equal allocation over our rolling study implementation period. We used the website Sealed Envelope for randomization (https://www.sealedenvelope.com/simple-randomiser/v1/lists). Following randomization, a research assistant emailed students the study instructions, including a link to their assigned module. All other team members were blinded to participants’ allocation. Within 24 hours of randomization, the study coordinator (AGG) mailed students a 12-page notebook. Students were instructed to complete the module in one sitting at their convenience, ideally within a couple days of receiving the notebook. Students could choose how much time to spend completing the module. If students did not complete the module within ten days of receiving the link, they were sent a reminder email. Seven days after they completed the module, students were sent a link to a post-module quiz. After completing the quiz, students provided re-consent and received a $25 gift card. Data collection occurred between August and December 2021.

### Outcome measures

#### Autonomous and controlled motivation

To start the module, students clicked through the following slides: title, introduction, life goal framing prompt (prompt condition only), and learning objectives. Thereafter, students completed an embedded ‘module survey’ that included an autonomous and controlled motivation questionnaire. Autonomous and controlled motivation were measured using the 16-item Self-Regulation Questionnaire – Academic (SRQ-A) [34]. We adapted items slightly to reflect the study context (e.g., replacing ‘parents’ as external agents with ‘the researchers’). We asked a researcher experienced in using the SRQ-A among medical students, who was not a co-author, to review and approve our adaptations. We presented response options on a 5-point Likert scale with anchors of *completely not important* and *very important* on the endpoints. The unstandardized Cronbach’s alphas for the autonomous and controlled motivation subscales were 0.90 (95% CI: 0.87–0.93) and 0.81 (95% CI: 0.77–0.86), respectively.

#### Learning strategy use

We adopted two established approaches for measuring students’ learning strategy use: (1) through their note-taking, and (2) through their engagement with features of the module [35,36].

First, we mailed students a 12-page notebook. Students mailed their notebook back immediately after completing the module, using a pre-paid envelope. We split the notebook into two sections and instructed students to: (1) use the initial eight blank pages to *help them learn the material*, and (2) use the 10 self-test questions (printed on the last four pages) to *help them check their understanding*. Students were free to use (or not use) the notebook as they saw fit. We quantified students’ organizational note-taking by summing the number of idea units from the module that they recorded in their notebook [35]. Using White and Mayer’s (1980) approach, two team members independently read through the full-text of the module and identified individual idea units. Disagreements were resolved through discussion, leading to a final list of 226 idea units. We adopted a *master coder* approach whereby one team member coded all of the data while another coder (the ‘reliability coder’) independently coded a subset of the data to establish inter-rater reliability [37]. The reliability coder coded 21 randomly selected notebooks (18%), a typical proportion [37,38]. The intraclass correlation (ICC) for organization strategy use was 0.99 (95% CI: 0.986 – 0.998). We quantified students’ elaborative note-taking by summing the number of examples, inferences, critical comments, and references to prior knowledge that they recorded in their notebook [35]. The ICC for elaboration strategy use was 0.95 (95% CI: 0.88 – 0.98). We quantified students’ metacognitive notetaking by summing the number of self-test questions that they answered. Nearly all (90%) students answered zero questions or ten questions, so we opted to dichotomize this variable: students answering one or more questions (strategy users) or answering zero questions (strategy non-users). We attempted to model this outcome in several other ways, none of which appreciably altered the results.

Second, we programmed the module to automatically count how many times students interacted with three features: embedded interactive sections (i.e., the number of times students clicked to access the interactive features), embedded questions (i.e., the number of times students clicked to reveal the answers to questions), and embedded links (i.e., the number of times students clicked on hyperlinks). We propose that students may engage with these features to help them organize and elaborate on the material by: (1) actively manipulating it (for the interactive sections), (2) comparing answers with their own guesses (for the questions), and (3) seeking supplementary information (for the links) [36].

#### Knowledge retention

We assessed students’ knowledge retention using a 30-minute online quiz featuring three open-ended questions, which asked students to explain the physiological responses to weight loss and their clinical implications in as much detail as possible [39]. Two team members independently counted the number of correct idea units students recorded in their quiz responses (out of the 229 idea units). The master coder coded all quizzes while the reliability coder coded 24 randomly selected quizzes (19%). The ICC was 0.80 (95% CI: 0.60 – 0.91).

### Moderator measure

#### Perceived competence for learning (PCL)

Students’ PCL was measured on the module survey using the 4-item Perceived Competence for Learning Scale (PCLS) [40]. We adapted items slightly to reflect the study context. Response options were presented on a 7-point Likert scale with anchors of *not at all true* and *very true* on the endpoints. The unstandardized Cronbach’s alpha for the scale was 0.86 (95% CI: 0.82 – 0.90).

### Control measures

#### Prior knowledge

We measured students’ prior knowledge on the module survey to control for in our analyses, given prior knowledge can influence how learners self-regulate their learning [41]. We followed Mayer and colleagues’ checklist approach [39], asking students to indicate whether three statements regarding their experience with the topic were true (e.g., “I know what the term metabolic adaptation means”). We selected this approach to reduce participant burden in reporting their prior knowledge, relative to a pre-test that corresponded to our knowledge retention quiz design. Only two participants (2%) answered affirmatively to one of the three statements, so it was dropped. Students were coded as *no prior knowledge* if they did not answer yes to any questions, *some prior knowledge* if they answered yes to one question, and *high prior knowledge* if they answered yes to both questions.

#### Initial interest

Research suggests that PCL positively correlates with interest ratings, so we measured initial interest on the module survey to account for in our analyses [29]. We adapted a single item to measure interest [31]. Response options were presented on a 7-point Likert scale with anchors of *not at all interesting* and *very interesting* on the endpoints.

### Statistical analyses

We conducted our analyses under a Bayesian framework. Bayesian regression models estimate a *posterior probability distribution* for each parameter in a model, which reflects the relative probabilities that a parameter takes on certain values, given the data and prior expectations [42]. Using probability distributions, researchers can calculate the probability that a parameter value lies above zero (i.e., is associated with benefit), and the range within which a specified percentage (e.g., 89%) of the most probable values lie, called a *highest density interval* (HDI). This information cannot be calculated from a Frequentist regression model, which is why we opted to conduct our analyses under a Bayesian framework.

A Bayesian regression model was constructed for each outcome, totaling nine models (see model parameterizations in Supplemental Digital Appendix 2). The priors for each model were intended to be weakly informative, meaning that they did not favour a particular response distribution prior to observing the actual data. Prior predictive checks provided visual evidence that our prior specifications could generate nearly every possible response distribution for each outcome, confirming that they were weakly informative.

The initial quantity of interest in answering Research Question #1 (i.e., whether the intervention had an effect) was the condition term in each model, and the quantity of interest in answering Research Question #2 (i.e., whether the effect of the prompt differed by PCL) was the interaction term between condition and PCL.^1^ Exclusion of zero in the 89% HDI was taken as an indication that the parameter was non-zero (i.e., statistically significant) [43]. Regression summary tables for our final models are included in Supplemental Digital Appendix 3. A condition term around zero in the presence of a significant interaction term suggests that the intervention may have different effects at different values of PCL. Therefore, if the interaction term in an outcome model was statistically significant, we did not use the condition term to determine whether the intervention had an effect. Rather, we used the joint posterior probability distribution to estimate the posterior predictive distribution for the difference in means between the prompt and no-prompt conditions. We calculated a separate group difference among students at three different levels of latent PCL: *low PCL* (less than one standard deviation below the mean, which included 15.0% of the students), *mean PCL* (between one standard deviation below and above the mean, which included 69.7% of the students), and *high PCL* (greater than one standard deviation above the mean, which included 15.3% of the students; [29]). We then calculated the 89% HDI for each group difference. Exclusion of zero in the 89% HDI was taken as an indication that the difference was non-zero.

Models were constructed using the brms and cmdstanr packages [44], as interfaces to the Stan programming language [45] in R statistical computing software (version 4.3.0) [46]. All parameters in all models had a scale reduction factor Rˆ of 1.00, providing evidence that the MCMC chains converged [47].

## Results

Of the 173 students who provided informed consent, 134 completed the module while 39 did not complete the module after multiple reminder emails. Six students completed the module but did not provide reconsent. Therefore, the complete study sample includes 128 students, with 65 randomized to the prompt condition and 63 randomized to the no-prompt condition (see Table 1 for participant demographics). Among students in the prompt condition, 59 (91%) had data regarding their typed reflection to the prompt. Among this group, 41 students (69%) typed a reflection while 18 students (31%) did not. Examples of reflections are included in Supplemental Digital Appendix 4. Descriptive plots of student responses across outcomes (and PCL) are included in Supplemental Digital Appendix 5.

**Table 1:** Participant demographics by condition.


DEMOGRAPHIC CHARACTERISTIC	PROMPT CONDITION	NO-PROMPT CONDITION

Age		

*≤23*	35 (48%)	38 (52%)

*>23*	30 (55%)	25 (45%)

Gender		

*Female*	52 (57%)	40 (43%)

*Male*	13 (37%)	22 (63%)

*Prefer Not to Say*	0 (0%)	1 (100%)

Intended Specialty		

*Family Medicine*	6 (50%)	6 (50%)

*Other*	28 (58%)	20 (42%)

*Unsure*	31 (46%)	37 (54%)

Expected Year of Medical School Graduation		

*2023*	9 (43%)	12 (57%)

*2024*	35 (56%)	27 (44%)

*2025*	21 (47%)	24 (53%)


### Effect of life goal framing on motivation

For autonomous motivation, the estimated effect of condition was 0.06 (89% HDI: -0.25 – 0.37) and the estimated interaction effect between condition and PCL was 0.25 (89% HDI: 0.02 – 0.47). The HDI surrounding the interaction effect does not include zero and can be considered statistically significant. Table 2 presents a summary of the posterior predictive distributions for the difference in means at low, mean, and high levels of PCL. The differences are not statistically significant at any level of PCL. For controlled motivation, the estimated effect of condition and the estimated interaction effect were not statistically significant (see Supplemental Digital Appendix 3).

**Table 2:** Summary of predicted probability distributions for group differences in autonomous motivation at different levels of PCL.


LEVEL OF PCL	MEAN	PROBABILITY > 0	89% HDI

Low	–0.32	0.05	–0.61–0.03

Mean	0.02	0.56	–0.18–0.19

High	0.29	0.92	–0.07–0.58


### Effect of life goal framing on learning strategy use

For engagement with links, the estimated effect of condition was 1.93 (89% HDI: 1.19 – 3.08), which can be interpreted as students in the prompt group clicking, on average, 93% more links than students in the control group. However, the absolute magnitude of this effect is quite small, as there was relatively little link-clicking among students overall (see Supplemental Digital Appendix 5). The estimated interaction effect between condition and PCL was not statistically significant. For all other outcomes, the estimated effect of condition and the estimated interaction effect were not statistically significant.

### Effect of life goal framing on knowledge retention

For knowledge retention, the estimated effect of condition and the estimated interaction effect were not statistically significant.

## Discussion

We investigated the effects of a life goal framing prompt on medical students’ autonomous and controlled motivation, learning strategy use, and knowledge retention, and the moderating effect of students’ PCL. The life goal framing prompt consisted of a single slide encouraging students to connect what they were learning to the life goals they pursue through a career in medicine.

Regarding Research Question #1, we did not find any statistically significant differences between the prompt and no-prompt conditions for any outcome (i.e., autonomous motivation, controlled motivation, organizational note-taking, elaborative note-taking, metacognitive note-taking, engagement with interactive sections, engagement with questions, knowledge retention), except for a small effect on link-clicking. Therefore, we cannot at this time conclude that prompting life goal framing is an effective strategy for enhancing medical students’ autonomous motivation, learning strategy use, and knowledge retention, even when they have high confidence. However, we suggest that there is plenty of room to strengthen the effect of a prompt. We suspect that the effect of the prompt was attenuated because nearly one-third of students randomized to receive the prompt did not provide a typed reflection, and thus may not have engaged in life goal framing in the desired manner [48]. The effect of a motivational stimulus depends on the degree to which learners process the stimulus in the intended manner [48]. We suspect that the students who did not provide a typed reflection did not process the prompt in the desired manner, either skipping over the prompt without reading it, or only superficially scanning the prompt. If some students did not process the prompt in the intended manner, then the effect of the prompt on autonomous motivation was likely attenuated, relative to the effect that would have been observed had all students processed the prompt in the intended manner. Supporting this view, studies have found that motivational interventions can yield attenuated effect sizes when they are implemented in naturalistic versus lab settings [49], and that learners demonstrate variable engagement with motivational stimuli in naturalistic settings [50,51]. Future research could investigate ways to enhance students’ engagement with the prompt. Future research could also focus on gaining a better understanding of the psychological process through which learners connect what they are learning to their life goals, toward designing more effective prompts.

That said, we did find a statistically significant interaction effect between condition and PCL on autonomous motivation, albeit a small effect. This interaction effect is consistent with our theoretical account of motivated engagement in learning, based on Control Theory and SDT. When learners perceive an activity as helping them accomplish their personally meaningful goals, their resultant autonomous motivation seems to *also* depend on their confidence that they can learn the presented material. This finding is consistent with some [29,52], but not all [31], prior research. According to Hecht and colleagues (2020), learners’ confidence may be particularly influential when learning a new skill, perhaps because skills need to be executed correctly to be useful, compared to when learning new concepts, as even partial understanding may be useful. For example, Hecht and colleagues (2020) found that undergraduate students’ PCL did not moderate the effects of a prompt when they studied the biology of fungi, whereas other studies have observed a moderating effect when students learned new math skills [29,52]. Given our prompt emphasized the utility of skillfully managing patients’ weight loss attempts, we suggest our results align with prior studies focusing on skill development [29,52].

By contrast, we did not find evidence for an interaction effect between condition and PCL on learning strategy use or knowledge retention. It could be that students with low *initial* PCL (i.e., when it was measured) developed higher PCL as they progressed through the module (or vice versa). If this was the case, initial PCL would be less predictive of their learning strategy use during the module (and knowledge retention following the module). Future research could test this interpretation by administering the SRQ-A and PCLS to students at several points during the module [53].

### Implications for education

Our findings add to a growing literature in HPE on design strategies for supporting learners’ motivation when learning online [14,15,16,54]. We appear to be the first in HPE to attempt to enhance learners’ motivation by helping them connect an activity to their future goals, building on recommendations that this might be an effective strategy [55]. At this time, we cannot recommend prompting life goal framing to educators as an effective design strategy, even when learners have high confidence. However, we anticipate that future research will identify more effective ways to prompt life goal framing.

Our study does suggest that, when learners make meaning of instructional activities, they do so in terms of their future goals *and* their present confidence. As such, when learners are not confident that they can successfully learn the target skills, prompting them to construe instruction in a more meaningful light may not be a productive strategy. Accordingly, we believe educators must consider learners’ confidence when they are learning online. Research suggests that providing learners with periodic, constructive feedback regarding their performance, in the context of digital games and online quizzes [16,56], can enhance their motivation, potentially by supporting their confidence.

### Limitations

By using a convenience sampling approach, our participants may have been more autonomously motivated relative to the broader student population. To limit this threat, we tried to prevent students from being disproportionately motivated by the *topic* by blinding them to the topic until they accessed the module. We used a self-report approach to capture prior knowledge, which may not have completely reflected students’ actual understanding of the physiology of weight loss. Students were not explicitly told not to collaborate with one another while completing the module, which may have risked contamination across study conditions. These limitations are balanced by our study’s strengths. To enhance authenticity, we allowed participants to complete the module at a time and place of their choosing, approximating the circumstances under which students typically complete online modules. Further, we used trace-based measures of strategy use, which tend to more accurately reflect self-regulatory behaviours compared to self-report measures [57]. We assessed knowledge with a delayed retention measure, which may be more sensitive to deep strategy use than an immediate post-test [58]. Finally, we controlled for potential moderator-outcome confounding by capturing initial interest [59].

## Conclusions

As learners navigate their training, they may over-emphasize how instruction helps them prepare for upcoming assessments, rather than how it provides them with the tools to become the professional (and person) they want to be. In this study, we tested a novel design strategy to help motivate students by leveraging the motivational energy associated with their life goals. We aim for this foundational study and our broader research program to motivate researchers to clarify how relatively simple interventions can help learners to connect their daily online work to their most meaningful goals.

## Additional Files

The additional files for this article can be found as follows:

10.5334/pme.1017.s1 Supplemental Digital Appendix 1. Text of the life goal framing prompt.

10.5334/pme.1017.s2 Supplemental Digital Appendix 2. Final parameterizations for Bayesian regression models.

10.5334/pme.1017.s3 Supplemental Digital Appendix 3. Regression summary tables for final Bayesian regression models.

10.5334/pme.1017.s4 Supplemental Digital Appendix 4. Examples of student responses to the prompt.

10.5334/pme.1017.s5 Supplemental Digital Appendix 5. Plots of student responses on study outcomes.
