# Supplementary material for: Effectiveness of Life Goal Framing to Motivate Medical Students During Online Learning: A Randomized Controlled Trial
Source: Perspect Med Educ. 2023 Oct 26;12(1):444–54. doi: 10.5334/pme.1017 (PMC10607565; doi:10.5334/pme.1017)
Supplement: Supplemental Digital Appendix 1. — Text of the life goal framing prompt. [file pme-12-1-1017-s1.pdf]

Before continuing, please take a moment to consider why it might be important for you to learn about the physiology of weight loss, including how this knowledge might prepare you to practice medicine in a way that is consistent with your own values and long-term goals.

One reason why it might be important is because it will prepare you to help patients with obesity become healthier and happier. Many patients with obesity report being motivated to reduce the risk for adiposity-related disease so they can actively engage with their loved ones and enjoy life’s activities. Often this involves weight loss. However, patients typically face considerable challenges in losing weight and thus require ongoing support from physicians and other health professionals.

For example, consider this quote by [X]. [X] is a 71-year-old former public service worker who has been working with his physician and other health professionals over the past decade to manage his risk of adiposity-related disease. We wanted to share his quote with you to give you a sense of the impact your learning about this topic could have on your future patients.

“I was born in 1950 and I have been heavy all my life. In January 2011 my physician told me that at 380 pounds, I had a 25% change of a heart attack or stroke in the next few years. As a result, over the next 4 years I participated in a weight management program offered by the Nova Scotia Health Authority. I was helped by my physician, dietitians, kinesiologists, and psychologists. All were integral to the changes I made. I ended up losing 110 pounds! With continued support by my physician, I currently maintain a weight of 270 pounds.

Over the course of losing weight I’ve rediscovered my love for woodworking because its easier for me to spend time on my feet now. I’ve even built a woodworking shop in my basement!

Without the knowledge and support of my physician and other health professionals, I doubt I would have achieved such positive results”.

By learning about the physiology of weight loss, you will be prepared to counsel patients in realistic, sustainable weight loss attempts to support their long-term health and happiness. How does this outcome align with the kind of physician you want to be? How does this outcome align with what you want to accomplish through being a physician? Jot down some of your thoughts in the text box below:

~ text box here ~

Thank you for jotting down some of your thoughts. You may find some aspects of this module challenging or boring. However, we hope that you persist because in the end you will have learned something that could have a significant impact on the lives of your future patients.
