# Supplementary material for: Effectiveness of Life Goal Framing to Motivate Medical Students During Online Learning: A Randomized Controlled Trial
Source: Perspect Med Educ. 2023 Oct 26;12(1):444–54. doi: 10.5334/pme.1017 (PMC10607565; doi:10.5334/pme.1017)
Supplement: Supplemental Digital Appendix 2. — Final parameterizations for Bayesian regression models. [file pme-12-1-1017-s2.pdf]

Autonomous and controlled motivation[a]	$p\left(y_i \text{Competency} = k \mid \mu_i, \sigma, \{\theta_j\}\text{z}\right) = \Phi\left((\theta_k - \mu_i)/\sigma\right) - \Phi\left((\theta_{k-1} - \mu_i)/\sigma\right)$
	$\mu_i = \eta_{Subject} + \eta_{Question} + \beta_0$
	$\eta_{Subject} = \sigma_{Subject} \times \zeta_{Subject}$
	$\sigma_{Subject} \sim \text{Normal}(0,2)$
	$\zeta_{Subject} \sim \text{Normal}(0,1)$
	$\eta_{Question} = \sigma_{Question} \times \zeta_{Question}$
	$\sigma_{Question} \sim \text{Normal}(0,1)$
	$\zeta_{Question} \sim \text{Normal}(0,1)$
	$\beta_0 = 0$
	$\theta_1, \ldots, \theta_{K-1} \sim \text{Normal}(0,4)$
	$p\left(y_{\text{Aut}} = k \mid \mu_{\text{Aut}}, \sigma_{\text{Aut}}, \{\theta_j\}\right) = \Phi\left((\theta_k - \mu_{\text{Aut}})/\sigma_{\text{Aut}}\right) - \Phi\left((\theta_{k-1} - \mu_{\text{Aut}})/\sigma_{\text{Aut}}\right)$
	$\mu = \eta^{\text{Aut}}_{Subject} + \eta^{\text{Aut}}_{Question} + \beta_0 + \beta_1 \text{Group}_{Intervention_i} + \beta_2 \eta_{Subject_i}$
	$+\beta_3 \left(\text{Group}_{Intervention_i} \times \eta_{Subject_i}\right) + \beta_4 \text{mo}(\text{Int}_i, \delta) + \beta_5 \text{PK}_{1,i} + \beta_6 \text{PK}_{2,i}$
	$\eta^{\text{Aut}}_{Subject} = \sigma^{\text{Aut}}_{Subject} \times \zeta^{\text{Aut}}_{Subject}$
	$\sigma^{\text{Aut}}_{Subject} \sim \text{Normal}(1,1)$
	$\zeta^{\text{Aut}}_{Subject} \sim \text{Normal}(0,1)$
	$\eta^{\text{Aut}}_{Question} = \sigma^{\text{Aut}}_{Question} \times \zeta^{\text{Aut}}_{Question}$
	$\sigma^{\text{Aut}}_{Question} \sim \text{Normal}(1,1)$
	$\zeta^{\text{Aut}}_{Question} \sim \text{Normal}(0,1)$
	$\beta_0 = 0$
	$\beta^{\text{Aut}}_{1,\ldots6} \sim \text{Normal}(0,2)$
	$\delta \sim \text{Dirichlet}(1)$
	$\sigma_{Aut} = 1/disc$
	$\log(disc) = \gamma_{[\text{Group}]}$
	$\gamma_{[\text{Group = Control}]} = 0$
	$\gamma_{[\text{Group = Intervention}]} \sim \text{Normal}(2,2)$
	$\theta_1, \ldots, \theta_{K-1} \sim \text{Normal}(0,4)$
	$p\left(y_{\text{Con}} = k \mid \mu_{\text{Con}}, \sigma_{\text{Con}}, \{\theta_j\}\right) = \Phi\left((\theta_k - \mu_{\text{Con}})/\sigma_{\text{Con}}\right) - \Phi\left((\theta_{k-1} - \mu_{\text{Con}})/\sigma_{\text{Con}}\right)$
	$\mu = \eta^{\text{Con}}_{Subject} + \eta^{\text{Con}}_{Question} + \beta_0 + \beta_1 \text{Group}_{Intervention_i} + beta_2 \eta_{Subject_i}$
	$+\beta_3 \left(\text{Group}_{Intervention_i} \times \eta_{Subject_i}\right) + \beta_4 \text{mo}(\text{Int}_i, \delta) + \beta_5 \text{PK}_{1,i} + \beta_6 \text{PK}_{2,i}$
	$\eta^{\text{Con}}_{Subject} = \sigma^{\text{Con}}_{Subject} \times \zeta^{\text{Con}}_{Subject}$
	$\sigma^{\text{Con}}_{Subject} \sim \text{Normal}(1,1)$
	$\zeta^{\text{Con}}_{Subject} \sim \text{Normal}(0,1)$
	$\eta^{\text{Con}}_{Question} = \sigma^{\text{Con}}_{Question} \times \zeta^{\text{Con}}_{Question}$
	$\sigma^{\text{Con}}_{Question} \sim \text{Normal}(1,1)$
	$\zeta^{\text{Con}}_{Question} \sim \text{Normal}(0,1)$
	$\beta_0 = 0$
	$\beta^{\text{Con}}_{1\ldots6} \sim \text{Normal}(0,2)$
	$\delta \sim \text{Dirichlet}(1)$
	$\sigma_{Con} = 1/disc$
	$\log(disc) = \gamma_{[\text{Group}]}$
	$\gamma_{[\text{Group = Control}]} = 0$
	$\gamma_{[\text{Group = Intervention}]} \sim \text{Normal}(2,2)$
	$\theta_1, \ldots, \theta_{K-1} \sim \text{Normal}(0,4)$
	$\begin{bmatrix} \eta^{\text{A}}_{\text{Subject}} \\ \eta^{\text{C}}_{\text{Subject}} \end{bmatrix} \sim \text{MVNormal}\left(\begin{bmatrix} 0 \\ 0 \end{bmatrix}, \mathbf{S}_{\text{Subject}}\right)$
	$\mathbf{S}_{\text{Subject}} = \begin{pmatrix} \sigma_{\eta^A_{\text{Subject}}} & 0 \\ 0 & \sigma_{\eta^C_{\text{Subject}}} \end{pmatrix} \mathbf{R}_{\text{Subject}} \begin{pmatrix} \sigma_{\eta^A_{\text{Subject}}} & 0 \\ 0 & \sigma_{\eta^C_{\text{Subject}}} \end{pmatrix}$
	$\begin{bmatrix} \eta^{\text{A}}_{\text{Question}} \\ \eta^{\text{C}}_{\text{Question}} \end{bmatrix} \sim \text{MVNormal}\left(\begin{bmatrix} 0 \\ 0 \end{bmatrix}, \mathbf{S}_{\text{Question}}\right)$
	$\mathbf{S}_{\text{Question}} = \begin{pmatrix} \sigma_{\eta^A_{\text{Question}}} & 0 \\ 0 & \sigma_{\eta^C_{\text{Question}}} \end{pmatrix} \mathbf{R}_{\text{Question}} \begin{pmatrix} \sigma_{\eta^A_{\text{Question}}} & 0 \\ 0 & \sigma_{\eta^C_{\text{Question}}} \end{pmatrix}$
	$\mathbf{R}_{\text{Subject}} \sim \text{LKJcorr}(1)$
	$\mathbf{R}_{\text{Question}} \sim \text{LKJcorr}(1)$

Organizational note-taking[b]	$,\sigma,\{\theta_j\}) = \Phi\big((\theta_k-\mu_i)/\sigma\big) - \Phi\big((\theta_{k-1}-\mu_i)/\sigma\big)$<br>$\mu_i = \eta_{Subject} + \eta_{Question} + \beta_0$<br>$\eta_{Subject} = \sigma_{Subject} \times \zeta_{Subject}$<br>$\sigma_{Subject} \sim \text{Normal}(0,2)$<br>$\zeta_{Subject} \sim \text{Normal}(0,1)$<br>$\eta_{Question} = \sigma_{Question} \times \zeta_{Question}$<br>$\sigma_{Question} \sim \text{Normal}(0,1)$<br>$\zeta_{Question} \sim \text{Normal}(0,1)$<br>$\beta_0 = 0$<br>$\ldots,\theta_{K-1} \sim \text{Normal}(0,4)$<br>$l_i\vert\mu_i,\phi) = \begin{cases} \xi + (1-\xi)\cdot\text{NegBin}(0\vert\mu_i,\phi) & \text{if } y_i = 0 \\ (1-\xi)\cdot\text{NegBin}(y_i\vert\mu_i,\phi) & \text{if } y_i \neq 0 \end{cases}$<br>$\xi = \beta_{0_\xi} + \beta_{1_\xi}\text{Group}_{Intervention_i} + \beta_{2_\xi}\text{mo}(\text{Int}_i,\delta) + \beta_{3_\xi}\text{PK}_{1,i} + \beta_{4_\xi}\text{PK}_{2,i}$<br>$\beta_{0_\xi} \sim \text{Normal}(0,2)$<br>$\beta_{1_\xi \ldots 6_\xi} \sim \text{Normal}(0,1)$<br>$\delta \sim \text{Dirichlet}(1)$<br>$\text{logit}(\phi) \sim \text{Gamma}(0.01,0.01)$<br>$\log(\mu_i) = \beta_0 + \beta_1\text{Group}_{Intervention_i} + \beta_2\eta_{Subject_i} + \beta_3\left(\text{Group}_{Intervention_i} \times \eta_{Subject_i}\right)$<br>$+\beta_4\text{mo}(\text{Int}_i,\delta) + \beta_5\text{PK}_{1,i} + \beta_6\text{PK}_{2,i}$<br>$\beta_0 \sim \text{Normal}(1,1)$<br>$\beta_{1\ldots6} \sim \text{Normal}(0,1)$<br>$\delta \sim \text{Dirichlet}(1)$
Elaborative note-taking[b]	$\mu_i,\sigma,\{\theta_j\}) = \Phi\big((\theta_k-\mu_i)/\sigma\big) - \Phi\big((\theta_{k-1}-\mu_i)/\sigma\big)$<br>$\mu_i = \eta_{Subject} + \alpha_{Question} + \beta_0$<br>$\eta_{Subject} = \sigma_{Subject} \times \zeta_{Subject}$<br>$\sigma_{Subject} \sim \text{Normal}(0,2)$<br>$\zeta_{Subject} \sim \text{Normal}(0,1)$<br>$\alpha_{Question} \sim \text{Normal}\big(\overline{\alpha}_{Question},\sigma_{Question}\big)$<br>$\overline{\alpha}_{Question} \sim \text{Normal}(0,1)$<br>$\sigma_{Question} \sim \text{Normal}(0,1)$<br>$\beta_0 = 0$<br>$_1,\ldots,\theta_{K-1} \sim \text{Normal}(0,4)$<br>$boration_\mu \sim \text{NegBin}(\mu_i,\phi)$<br>$\text{logit}(\phi) \sim \text{Gammma}(0.01,0.01)$<br>$\log(\mu_i) = \beta_0 + \beta_1\text{Group}_{Intervention_i} + \beta_2\eta_{Subject_i} + \beta_3\left(\text{Group}_{Intervention_i} \times \eta_{Subject_i}\right)$<br>$+\beta_4\text{mo}(\text{Int}_i,\delta) + \beta_5\text{PK}_{1,i} + \beta_6\text{PK}_{2,i}$<br>$\beta_0 \sim \text{Normal}(1,1)$<br>$\beta_{1\ldots6} \sim \text{Normal}(0,1)$<br>$\delta \sim \text{Dirichlet}(1)$

Metacognitive note-taking[b]	$\mu_i, \sigma, \{\theta_j\}) = \Phi\big((\theta_k - \mu_i)/\sigma\big) - \Phi\big((\theta_{k-1} - \mu_i)/\sigma\big)$<br>$\mu_i = \eta_{Subject} + \alpha_{Question} + \beta_0$<br>$\eta_{Subject} = \sigma_{Subject} \times \zeta_{Subject}$<br>$\sigma_{Subject} \sim \text{Normal}(0,2)$<br>$\zeta_{Subject} \sim \text{Normal}(0,1)$<br>$\alpha_{Question} \sim \text{Normal}(\overline{\alpha}_{Question}, \sigma_{Question})$<br>$\overline{\alpha}_{Question} \sim \text{Normal}(0,1)$<br>$\sigma_{Question} \sim \text{Normal}(0,1)$<br>$\beta_0 = 0$<br>$_1, \ldots, \theta_{K-1} \sim \text{Normal}(0,4)$<br>$cognitive_i \sim \text{Bernoulli}(p_i)$<br>$\text{logit}(p_i) = \beta_0 + \beta_1 \text{Group}_{Intervention_i} + \beta_2 \eta_{Subject_i} + \beta_3 \left(\text{Group}_{Intervention_i} \times \eta_{Subject_i}\right)$<br>$+ \beta_4 \text{mo}(\text{Int}_i, \delta) + \beta_5 \text{PK}_{1,i} + \beta_6 \text{PK}_{2,i}$<br>$\beta_0 \sim \text{Normal}(1,1)$<br>$\beta_{1 \ldots 6} \sim \text{Normal}(0,1)$<br>$\delta \sim \text{Dirichlet}(1)$
Engagement with interactive sections[c]	$, \sigma, \{\theta_j\}) = \Phi\big((\theta_k - \mu_i)/\sigma\big) - \Phi\big((\theta_{k-1} - \mu_i)/\sigma\big)$<br>$\mu_i = \eta_{Subject} + \eta_{Question} + \beta_0$<br>$\eta_{Subject} = \sigma_{Subject} \times \zeta_{Subject}$<br>$\sigma_{Subject} \sim \text{Normal}(0,2)$<br>$\zeta_{Subject} \sim \text{Normal}(0,1)$<br>$\eta_{Question} = \sigma_{Question} \times \zeta_{Question}$<br>$\sigma_{Question} \sim \text{Normal}(0,1)$<br>$\zeta_{Question} \sim \text{Normal}(0,1)$<br>$\beta_0 = 0$<br>$\ldots, \theta_{K-1} \sim \text{Normal}(0,4)$<br>$iments_i \sim \text{Poisson}(\lambda_i)$<br>$\log(\lambda_i) = \beta_0 + \beta_1 \text{Group}_{Intervention_i} + \beta_2 \eta_{Subject_i} + \beta_3 \left(\text{Group}_{Intervention_i} \times \eta_{Subject_i}\right)$<br>$+ \beta_4 \text{mo}(\text{Int}_i, \delta) + \beta_5 \text{PK}_{1,i} + \beta_6 \text{PK}_{2,i}$<br>$\beta_0 \sim \text{Normal}(1,1)$<br>$\beta_{1 \ldots 6} \sim \text{Normal}(0,1)$<br>$\delta \sim \text{Dirichlet}(1)$
Engagement with questions[c]	$, \sigma, \{\theta_j\}) = \Phi\big((\theta_k - \mu_i)/\sigma\big) - \Phi\big((\theta_{k-1} - \mu_i)/\sigma\big)$<br>$\mu_i = \eta_{Subject} + \eta_{Question} + \beta_0$<br>$\eta_{Subject} = \sigma_{Subject} \times \zeta_{Subject}$<br>$\sigma_{Subject} \sim \text{Normal}(0,2)$<br>$\zeta_{Subject} \sim \text{Normal}(0,1)$<br>$\eta_{Question} = \sigma_{Question} \times \zeta_{Question}$<br>$\sigma_{Question} \sim \text{Normal}(0,1)$<br>$\zeta_{Question} \sim \text{Normal}(0,1)$<br>$\beta_0 = 0$<br>$\ldots, \theta_{K-1} \sim \text{Normal}(0,4)$<br>$s_i \mid \mu_i, \phi) = \begin{cases} \xi + (1 - \xi) \cdot \text{Pois}(0 \mid \lambda_i) & \text{if } y_i = 0 \\ (1 - \xi) \cdot \text{Pois}(y_i \mid \lambda_i) & \text{if } y_i \neq 0 \end{cases}$<br>$\text{logit}(\xi) = \beta_{0_\xi} + \beta_{1_\xi} \text{Group}_{Intervention_i} + \beta_{2_\xi} \text{mo}(\text{Int}_i, \delta) + \beta_{3_\xi} \text{PK}_{1,i} + \beta_{4_\xi} \text{PK}_{2,i}$<br>$\beta_{0_\xi} \sim \text{Normal}(0,2)$<br>$\beta_{1_\xi \ldots 6_\xi} \sim \text{Normal}(0,1)$<br>$\log(\lambda_i) = \beta_0 + \beta_1 \text{Group}_{Intervention_i} + \beta_2 \eta_{Subject_i} + \beta_3 \left(\text{Group}_{Intervention_i} \times \eta_{Subject_i}\right)$<br>$+ \beta_4 \text{mo}(\text{Int}_i, \delta) + \beta_5 \text{PK}_{1,i} + \beta_6 \text{PK}_{2,i}$<br>$\beta_0 \sim \text{Normal}(1,1)$<br>$\beta_{1 \ldots 6} \sim \text{Normal}(0,1)$<br>$\delta \sim \text{Dirichlet}(1)$

Engagement with links[c]	$,\sigma,\{\theta_j\}) = \Phi((\theta_k - \mu_i)/\sigma) - \Phi((\theta_{k-1} - \mu_i)/\sigma)$<br>$\mu_i = \eta_{Subject} + \eta_{Question} + \beta_0$<br>$\eta_{Subject} = \sigma_{Subject} \times \zeta_{Subject}$<br>$\sigma_{Subject} \sim \text{Normal}(0,2)$<br>$\zeta_{Subject} \sim \text{Normal}(0,1)$<br>$\eta_{Question} = \sigma_{Question} \times \zeta_{Question}$<br>$\sigma_{Question} \sim \text{Normal}(0,1)$<br>$\zeta_{Question} \sim \text{Normal}(0,1)$<br>$\beta_0 = 0$<br>$\ldots, \theta_{K-1} \sim \text{Normal}(0,4)$<br>$Links_\mu \sim \text{NegBin}(\mu_i, \phi)$<br>$\text{logit}(\phi) \sim \text{Gamma}(0.01,0.01)$<br>$\log(\mu_i) = \beta_0 + \beta_1 \text{Group}_{Intervention_i} + \beta_2 \eta_{Subject_i} + \beta_3 \left(\text{Group}_{Intervention_i} \times \eta_{Subject_i}\right)$<br>$+\beta_4 \text{mo}(\text{Int}_i, \delta) + \beta_5 \text{PK}_{1,i} + \beta_6 \text{PK}_{2,i}$<br>$\beta_0 \sim \text{Normal}(1,1)$<br>$\beta_{1\ldots6} \sim \text{Normal}(0,1)$<br>$\delta \sim \text{Dirichlet}(1)$
Knowledge retention[d]	$\theta_j\}) = \Phi((\theta_k - \mu_i)/\sigma) - \Phi((\theta_{k-1} - \mu_i)/\sigma)$<br>$\mu_i = \eta_{Subject} + \eta_{Question} + \beta_0$<br>$_{bject} = \sigma_{Subject} \times \zeta_{Subject}$<br>$_{bject} \sim \text{Normal}(0,2)$<br>$_{bject} \sim \text{Normal}(0,1)$<br>$_{stion} = \sigma_{Question} \times \zeta_{Question}$<br>$_{stion} \sim \text{Normal}(0,1)$<br>$_{stion} \sim \text{Normal}(0,1)$<br>$\beta_0 = 0$<br>$_{K-1} \sim \text{Normal}(0,4)$<br>$uiz_\mu \sim \text{NegBin}(\mu_i, \phi)$<br>$(\phi) \sim \text{Gamma}(0.01,0.01)$<br>$(\mu_i) = \beta_0 + \beta_1 \text{Group}_{Intervention_i} + \beta_2 \eta_{Subject_i} + \beta_3 \left(\text{Group}_{Intervention_i} \times \eta_{Subject_i}\right)$<br>$+\beta_4 \text{mo}(\text{Int}_i, \delta) + \beta_5 \text{PK}_{1,i} + \beta_6 \text{PK}_{2,i}$<br>$\beta_0 \sim \text{Normal}(1,1)$<br>$_{1\ldots6} \sim \text{Normal}(0,1)$<br>$\delta \sim \text{Dirichlet}(1)$

[a]Questionnaire data was collected for 124 students (97%). We attributed missing data primarily to technical errors.

[b]Notebook-based data was collected for 114 students (89%). We attributed missing data primarily to notebooks being lost in the mail.

[c]Computer-based data was collected for 124 students (97%). We attributed missing data primarily to technical errors. An additional 5 students had incomplete computer-based data; these students were considered as missing computer-based data.

[d]Quiz data was collected for 125 students (98%). We did not administer quizzes to three students as it would have required them to complete the quiz during the winter holiday break. An additional 3 students opened the quiz link but did not progress to the quiz page; these students were considered quiz non-completers.
