# Supplementary material for: Effectiveness of Life Goal Framing to Motivate Medical Students During Online Learning: A Randomized Controlled Trial
Source: Perspect Med Educ. 2023 Oct 26;12(1):444–54. doi: 10.5334/pme.1017 (PMC10607565; doi:10.5334/pme.1017)
Supplement: Supplemental Digital Appendix 3. — Regression summary tables for final Bayesian regression models. [file pme-12-1-1017-s3.pdf]

Supplemental Digital Appendix 3. Regression summary tables for final Bayesian regression models.

Autonomous motivation

**Predictor**	**Difference in latent score (89% HDI)**
Condition	0.06 (-0.25 – 0.37)
PCL	0.02 (-0.16 – 0.20)
Condition*PCL	0.25 (0.02 – 0.47)
Interest	0.23 (0.11 – 0.37)
LowPriorKnowledge	-0.19 (-0.54 – 0.15)
HighPriorKnowledge	-0.31 (-0.68 – 0.05)
Random effects	
$\sigma_{Subject}$	1.05 (0.91 – 1.22)
σ_{Item}	0.67 (0.43 – 1.17)
Disc effects	
Condition	0.04 (-0.07 – 0.14)

Controlled motivation

**Predictor**	**Difference in latent score (89% HDI)**
Condition	-0.10 (-0.40 – 0.19)
PCL	0.00 (-0.16 – 0.16)
Condition*PCL	0.09 (-0.10 – 30.31)
Interest	-0.29 (-0.48 – -0.14)
LowPriorKnowledge	0.08 (-0.25 – 0.42)
HighPriorKnowledge	0.40 (0.05 – 0.74)
Random effects	
$\sigma_{Subject}$	0.99 (0.84 – 1.16)
σ_{Item}	1.28 (0.88 – 1.98)
Disc effects	
Condition	0.13 (-0.25 – -0.02)

Organizational note-taking

**Predictor (zi)**	**Relative probability (89% HDI)**

Condition	0.74 (0.59 – 0.85)
Interest	0.50 (0.42 – 0.58)
LowPriorKnowledge	0.27 (0.12 – 0.47)
HighPriorKnowledge	0.31 (0.14 – 0.53)
**Predictor**	**Relative count (89% HDI)**
Condition	1.09 (0.82 – 1.47)
PCL	1.01 (0.88 – 1.17)
Condition*PCL	0.83 (0.64 – 1.08)
Interest	1.11 (0.99 – 1.26)
LowPriorKnowledge	0.84 (0.59 – 1.21)
HighPriorKnowledge	0.74 (0.50 – 1.09)

Elaborative note-taking

**Predictor**	**Relative count (89% HDI)**
Condition	0.71 (0.32 – 1.54)
PCL	1.34 (0.87 – 2.12)
Condition*PCL	0.94 (0.30 – 2.70)
Interest	0.85 (0.64 – 1.11)
LowPriorKnowledge	0.87 (0.36 – 2.04)
HighPriorKnowledge	1.09 (0.45 – 2.71)

Metacognitive note-taking

**Predictor**	**Odds ratio (89% HDI)**
Condition	0.81 (0.43 – 1.52)
PCL	0.97 (0.66 – 1.45)
Condition*PCL	0.75 (0.37 – 1.41)
Interest	0.88 (0.67 – 1.13)
LowPriorKnowledge	1.38 (0.67 – 2.90)
HighPriorKnowledge	1.53 (0.72 – 3.23)

Engagement with questions

**Predictor (zi)**	**Relative probability (89% HDI)**
Condition	0.63 (0.44 – 0.78)
Interest	0.52 (0.44 – 0.6)
LowPriorKnowledge	0.53 (0.33 – 0.72)
HighPriorKnowledge	0.44 (0.24 – 0.65)
**Predictor**	**Relative count (89% HDI)**
Condition	0.96 (0.81 – 1.14)
PCL	0.98 (0.88 – 1.1)
Condition*PCL	1.00 (0.86 – 1.17)
Interest	1.06 (0.99 – 1.14)
LowPriorKnowledge	1.16 (0.93 – 1.44)
HighPriorKnowledge	1.22 (0.98 – 1.53)

Engagement with interactive sections

**Predictor**	**Relative count (89% HDI)**
Condition	1.04 (0.86 – 1.26)
PCL	0.98 (0.86 – 1.12)
Condition*PCL	1.06 (0.90 – 1.26)
Interest	1.05 (0.96 – 1.14)
LowPriorKnowledge	0.98 (0.77 – 1.23)
HighPriorKnowledge	0.91 (0.72 – 1.16)

Engagement with links

**Predictor**	**Relative count (89% HDI)**
Condition	0.66 (0.54 – 0.75)
PCL	1.07 (0.75 – 1.54)
Condition*PCL	0.90 (0.58 – 1.4)

Interest	1.06 (0.85 – 1.27)
LowPriorKnowledge	1.11 (0.65 – 1.86)
HighPriorKnowledge	0.66 (0.37 – 1.16)

Knowledge retention

**Predictor**	**Relative count (89% HDI)**
Condition	1.23 (0.69 – 2.22)
PCL	1.15 (0.79 – 1.73)
Condition*PCL	0.98 (0.57 – 1.74)
Interest	1.15 (0.94 – 1.44)
LowPriorKnowledge	0.87 (0.71 – 1.09)
HighPriorKnowledge	1.40 (0.73 – 2.62)
ExtraTime	1.03 (0.45 – 2.42)
Condition*ExtraTime	1.19 (0.36 – 4.00)
