# Supplementary material for: Effectiveness of Life Goal Framing to Motivate Medical Students During Online Learning: A Randomized Controlled Trial
Source: Perspect Med Educ. 2023 Oct 26;12(1):444–54. doi: 10.5334/pme.1017 (PMC10607565; doi:10.5334/pme.1017)
Supplement: Supplemental Digital Appendix 4. — Examples of student responses to the prompt. [file pme-12-1-1017-s4.pdf]

"I'd like to be an empathetic physician who patients can trust and have conversations about their health. Patients may look to me for direction for weight loss, and being able to understand the physiology of weight loss is going to be crucial to providing realistic, sustainable weight loss."

"The physician I hope to be is one that helps and supports their patients achieve their health goals, be it weight loss or reduction in A1C. [X]'s story is so inspiring. The physician involved had an immense impact on [X]'s life, and this is something that I hope to accomplish through my practice."

"I want to make a positive impact like the one provided in [X]'s story. It seems as if the physician was very supportive about his weight loss and played a strong factor in [X] obtaining his goal. Although physiologically challenging, weight loss in not impossible but does take a lot of work [sic]. When I become a physician, I want my words and actions to impact my patients ina positive want [sic]. By doing so, I will help reimagine what is possible for my patients."

"I am interested in providing support for patients in a way that makes them feel capable and in control of their future- so knowing how the physiology of weight loss functions is helpful, because it allows me to provide the most accurate information to patients. That way patients are aware of the struggles and the reality when they decide to make these changes, and I will be able to better support them through their journey"
