# Supplementary material for: Effectiveness of Life Goal Framing to Motivate Medical Students During Online Learning: A Randomized Controlled Trial
Source: Perspect Med Educ. 2023 Oct 26;12(1):444–54. doi: 10.5334/pme.1017 (PMC10607565; doi:10.5334/pme.1017)

Supplemental Digital Appendix 5. Plots of student responses on study outcomes.

Plot 1. Student responses on the autonomous motivation subscale of the Self-Regulation Questionnaire – Academic.

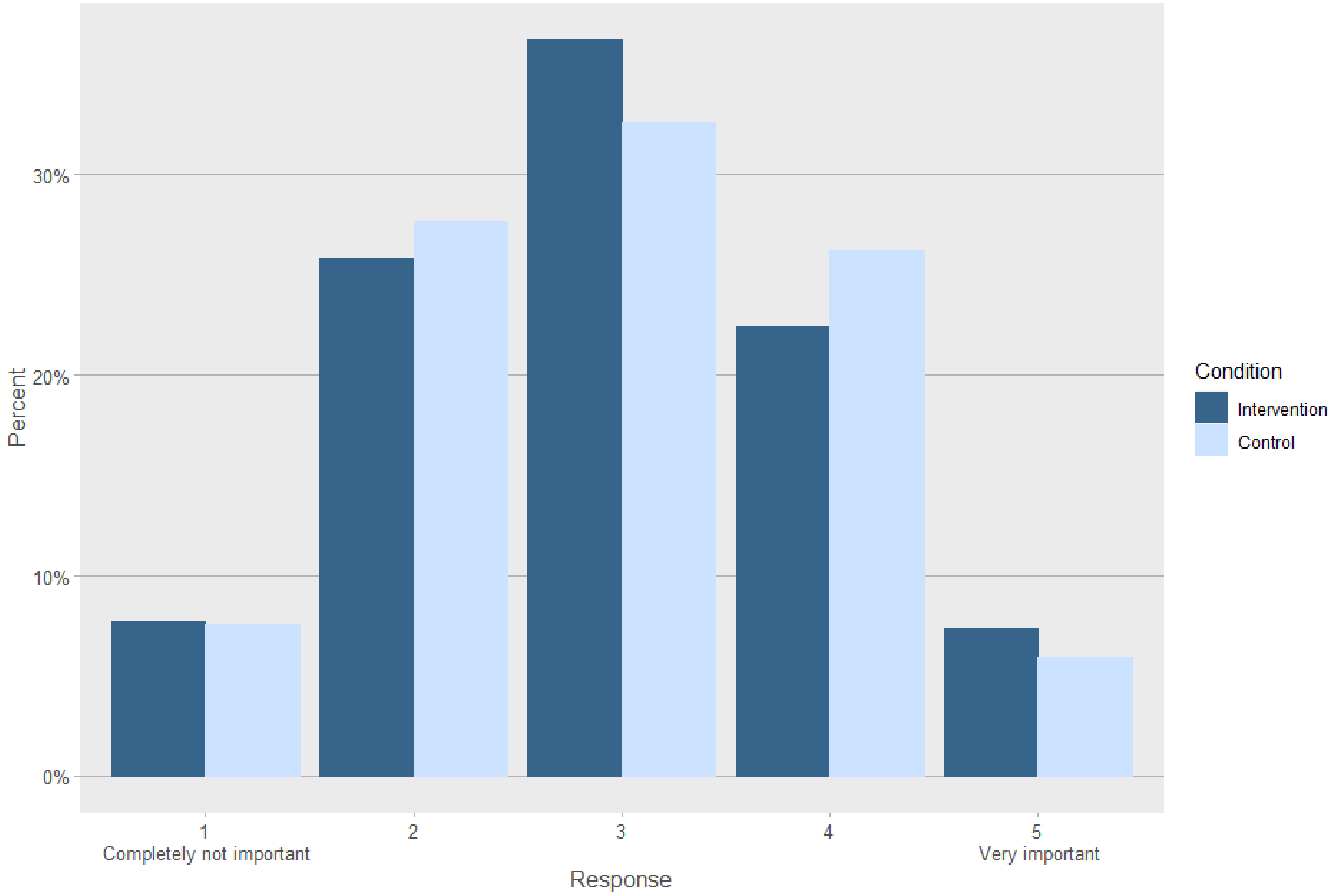

Plot 2. Student responses on the controlled motivation subscale of the Self-Regulation Questionnaire – Academic.

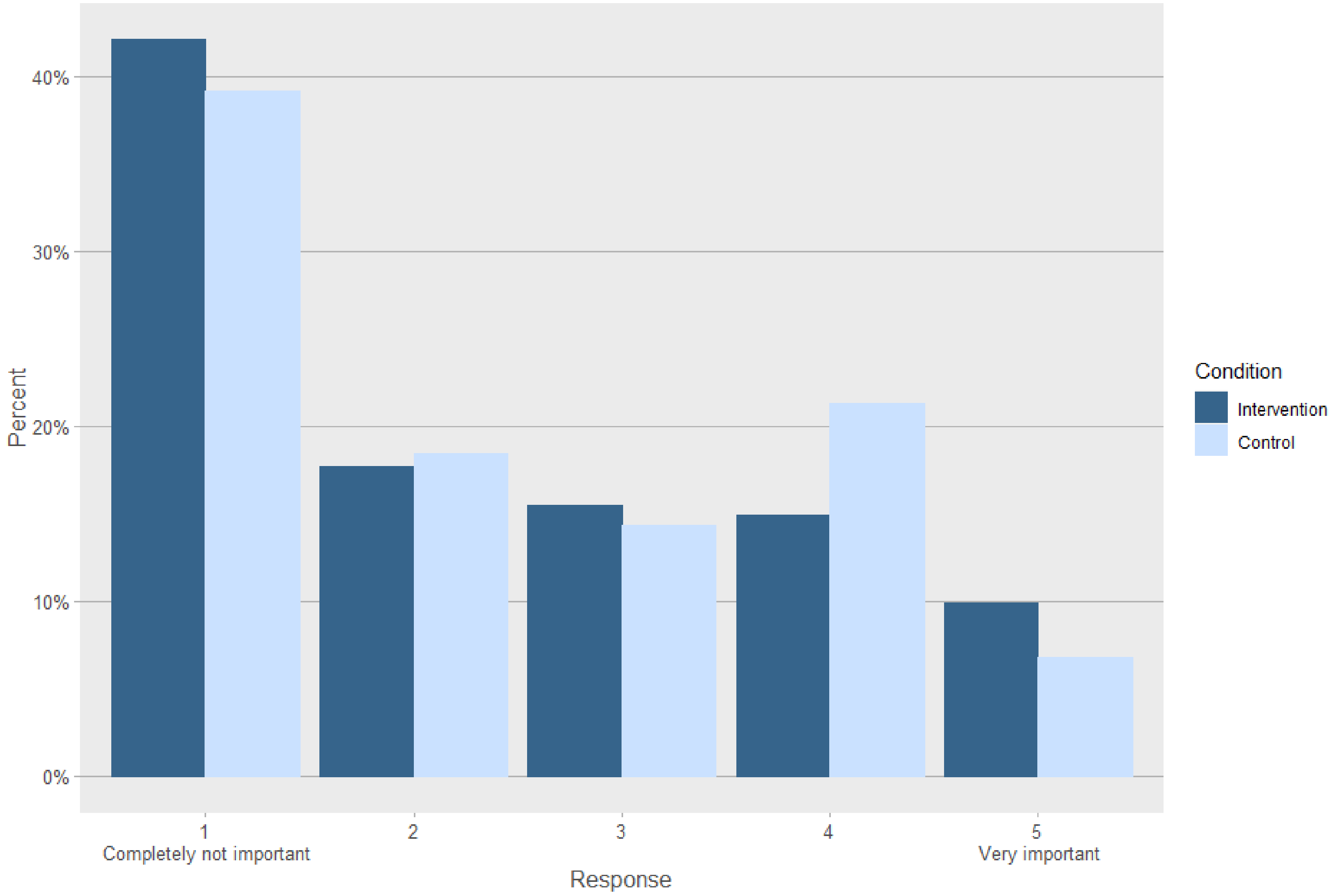

Supplemental Digital Appendix 5. Plots of student responses on study outcomes.

Plot 3. Student responses on the Perceived Competence for Learning Scale.

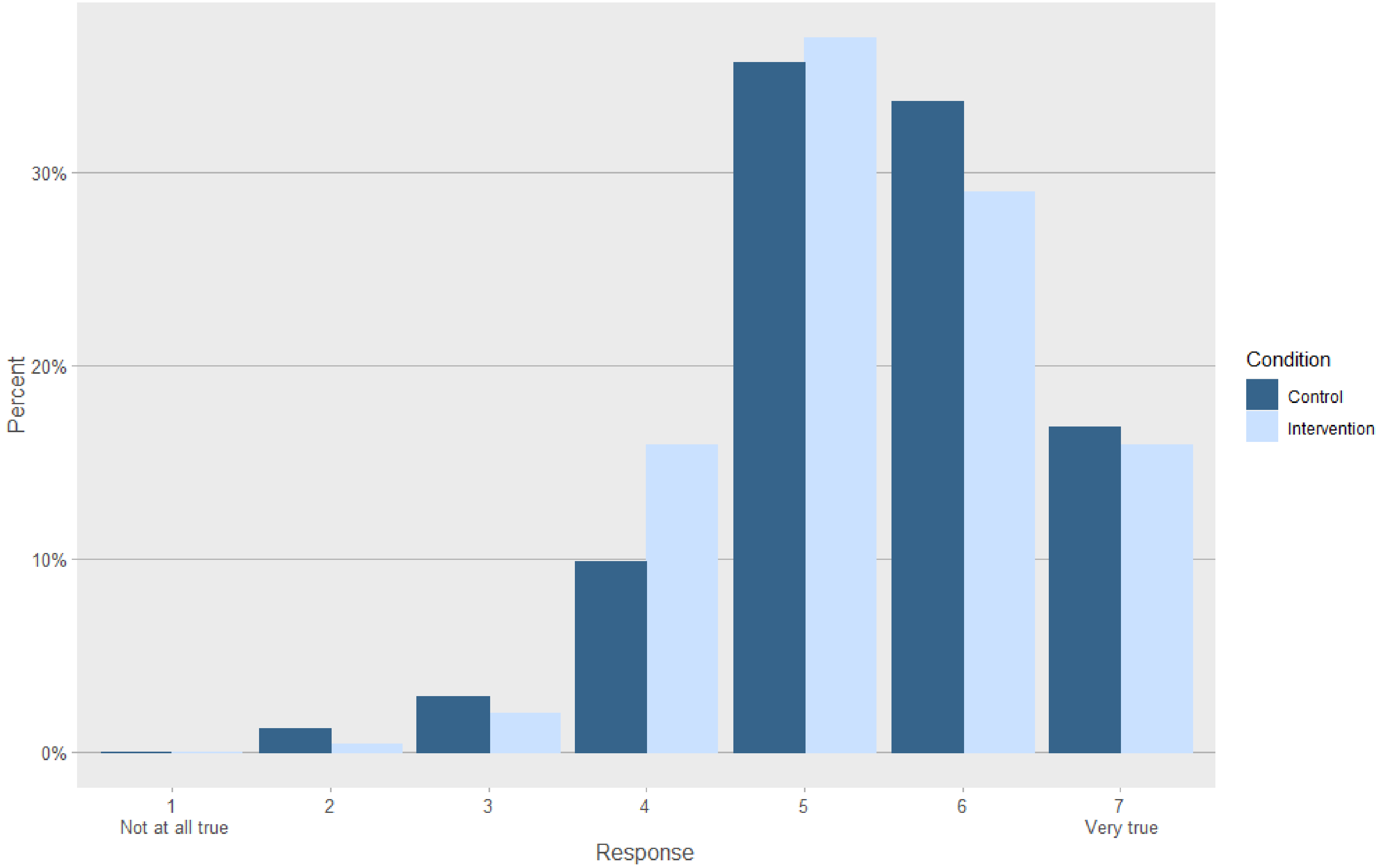

Supplemental Digital Appendix 5. Plots of student responses on study outcomes.

Plot 4. Student data regarding organizational note-taking.

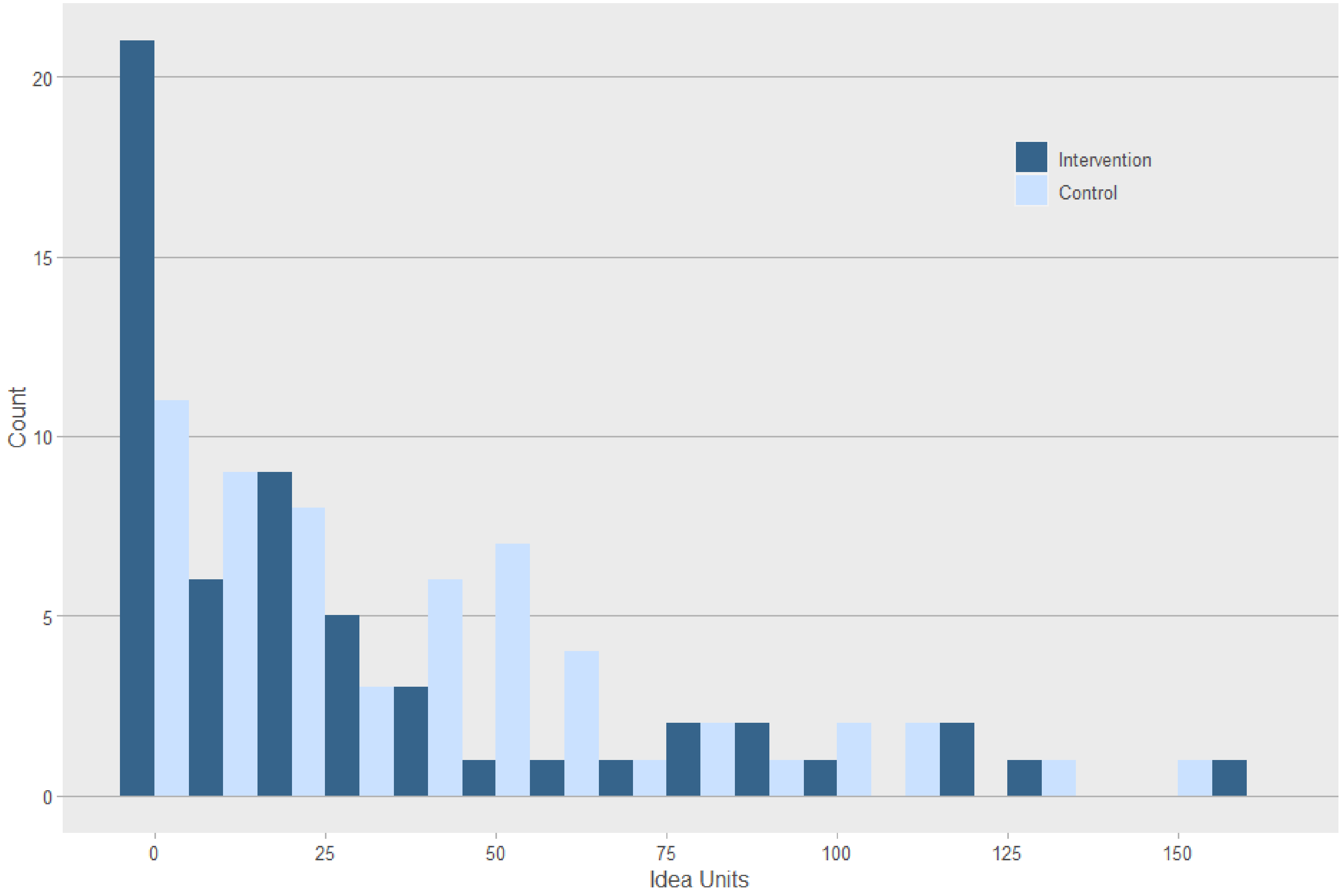

Plot 5. Student data regarding elaborative note-taking.

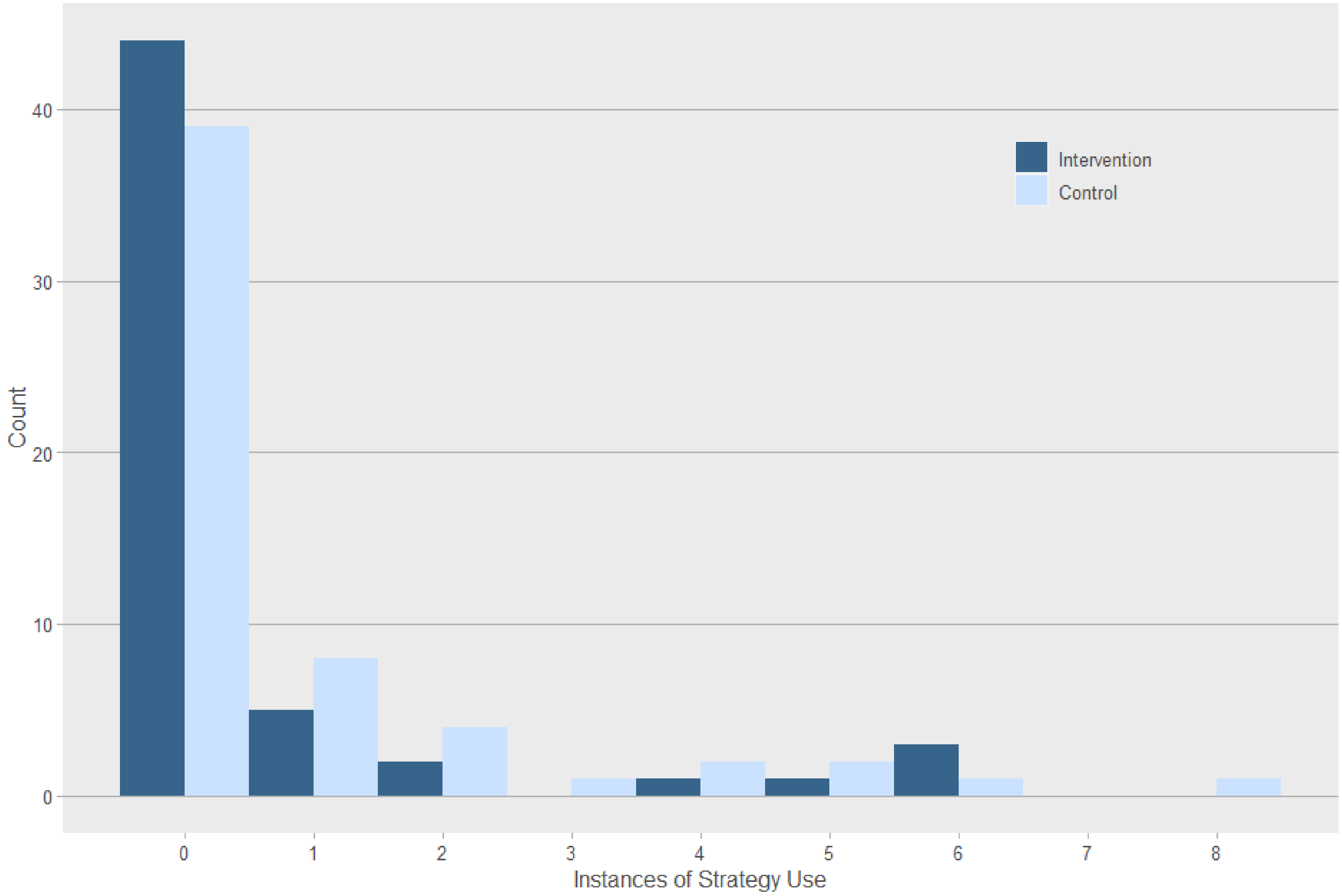

Plot 6. Student data regarding metacognitive note-taking.

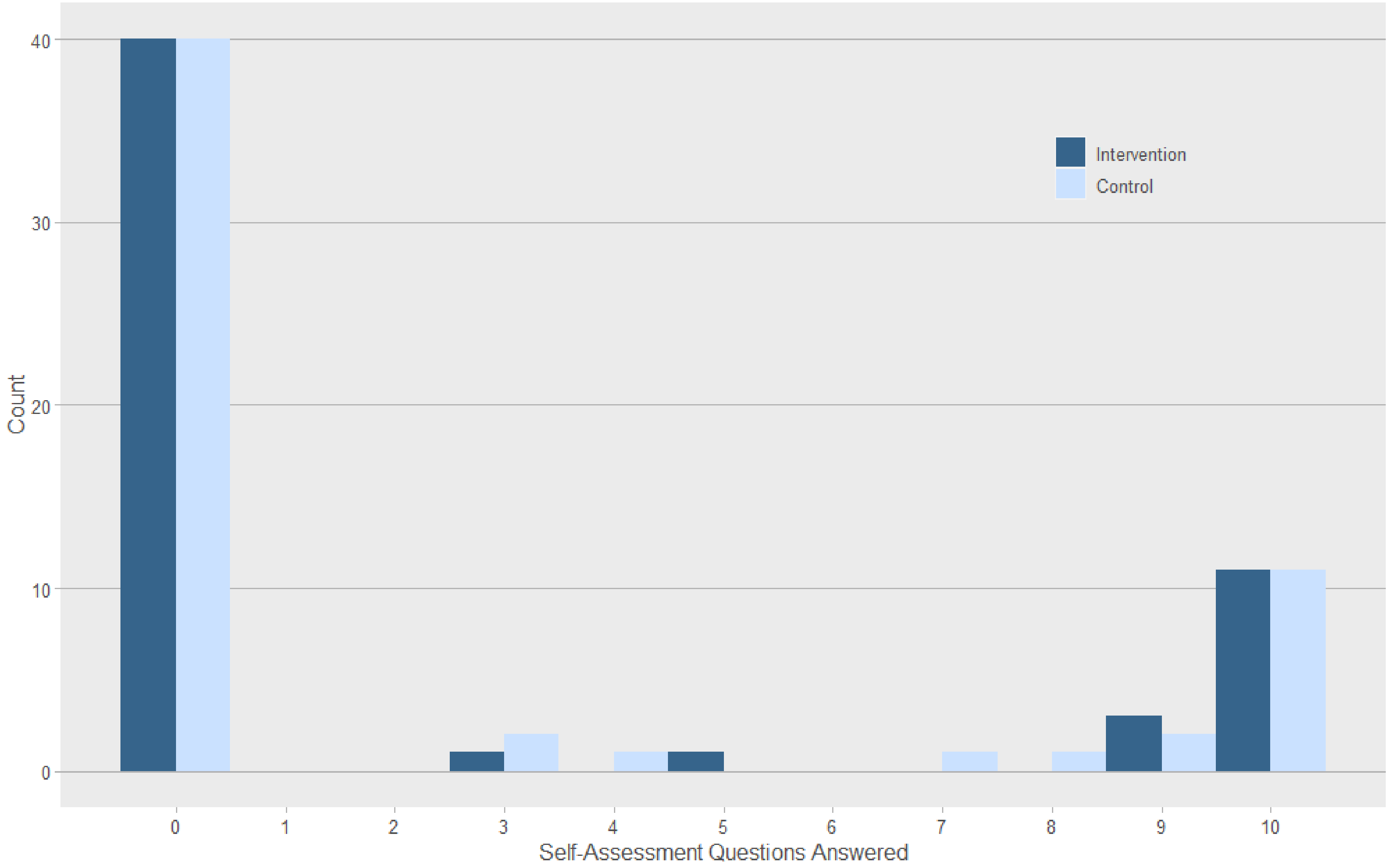

Plot 7. Student data regarding engagement with questions.

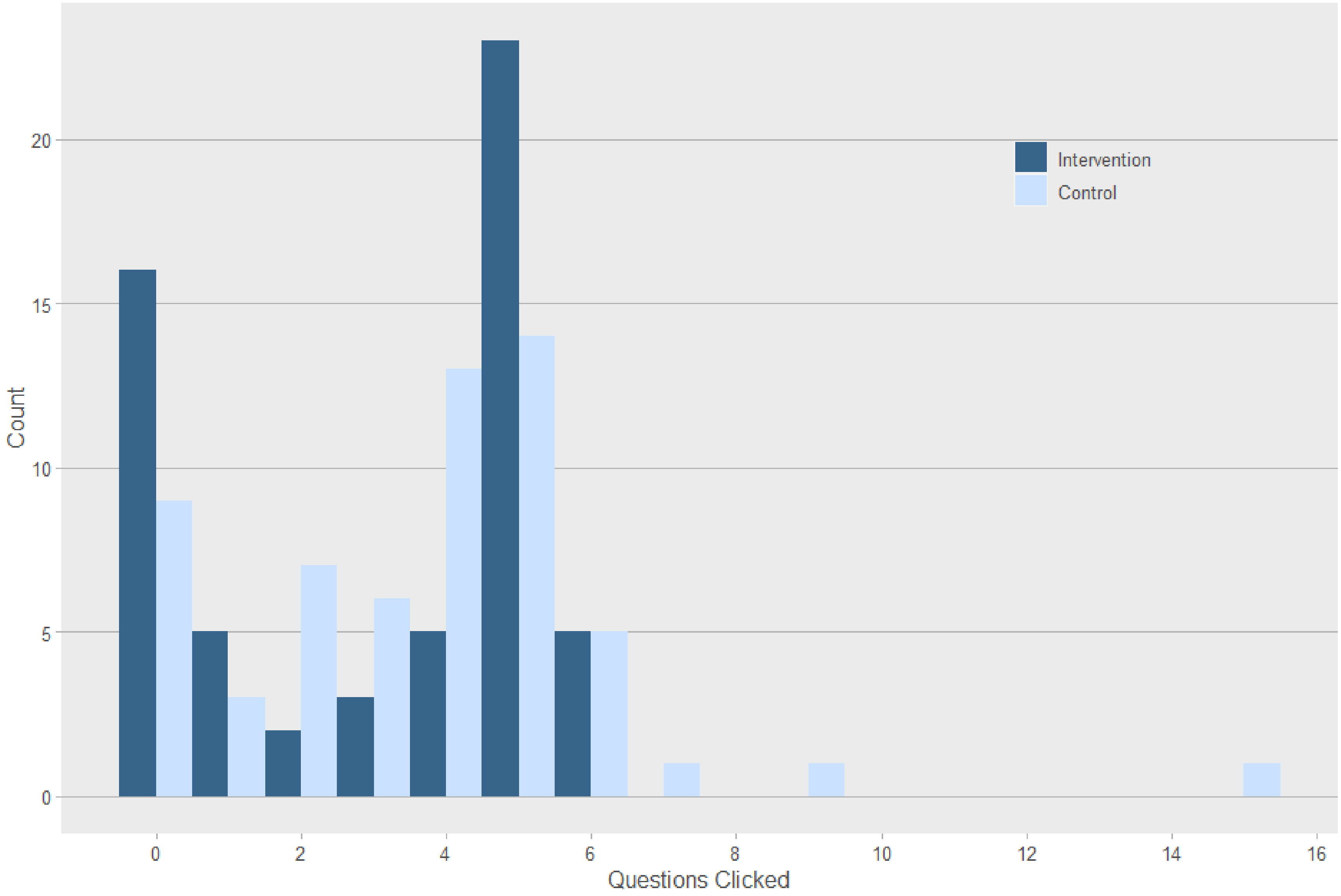

Plot 8. Student data regarding engagement with interactive sections.

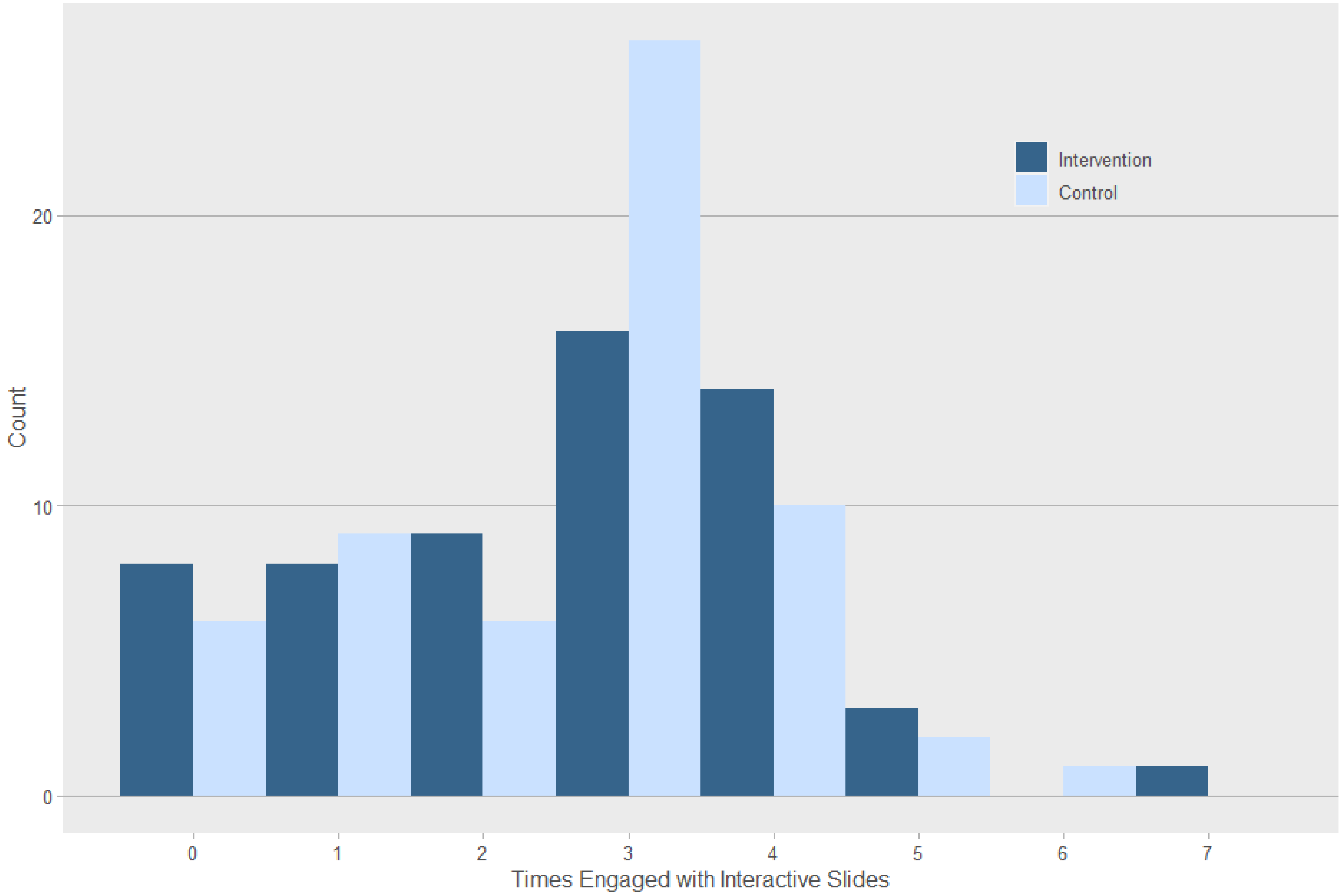

Supplemental Digital Appendix 5. Plots of student responses on study outcomes.

Plot 9. Student data regarding engagement with links.

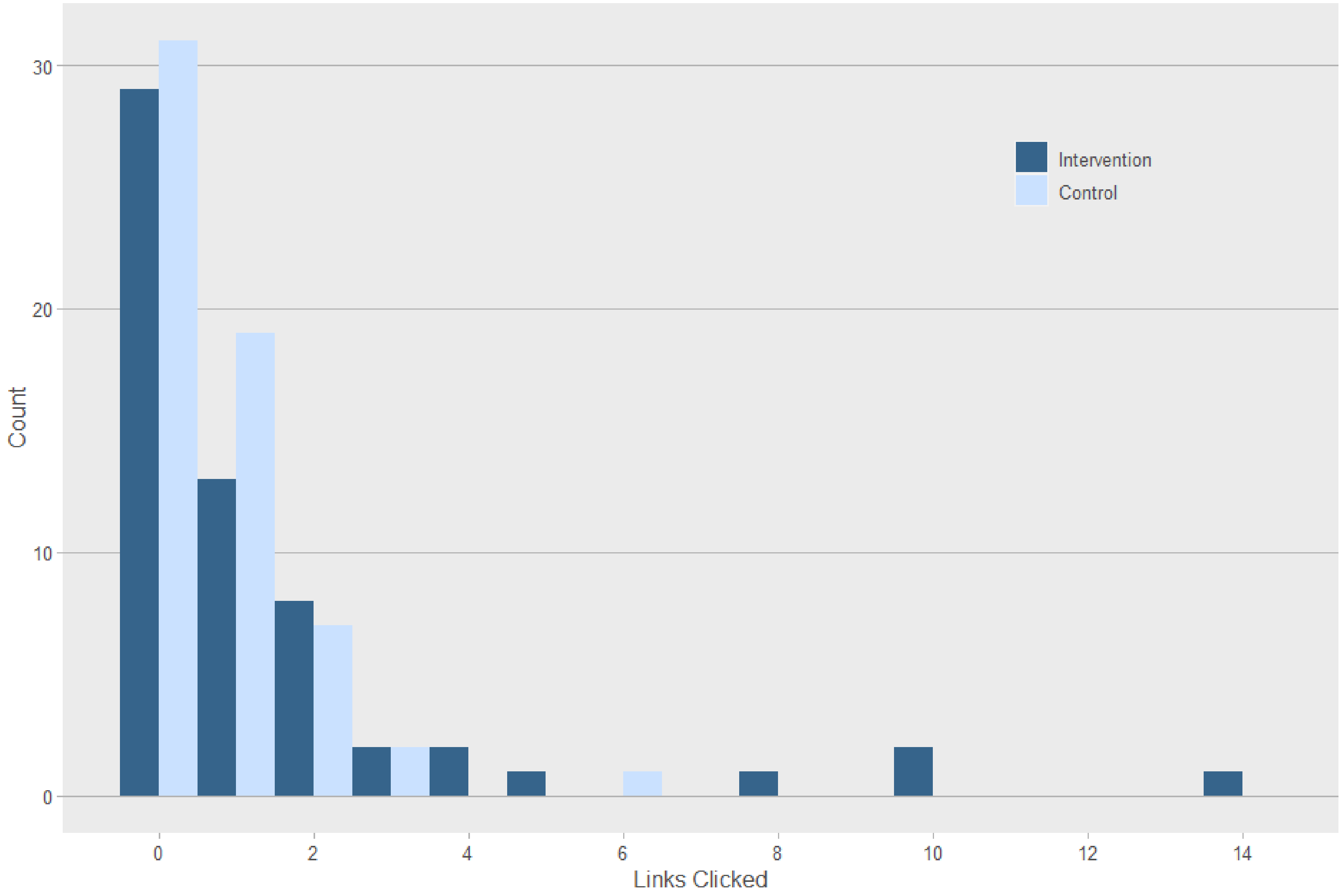

Plot 10. Student performance on the knowledge retention test.

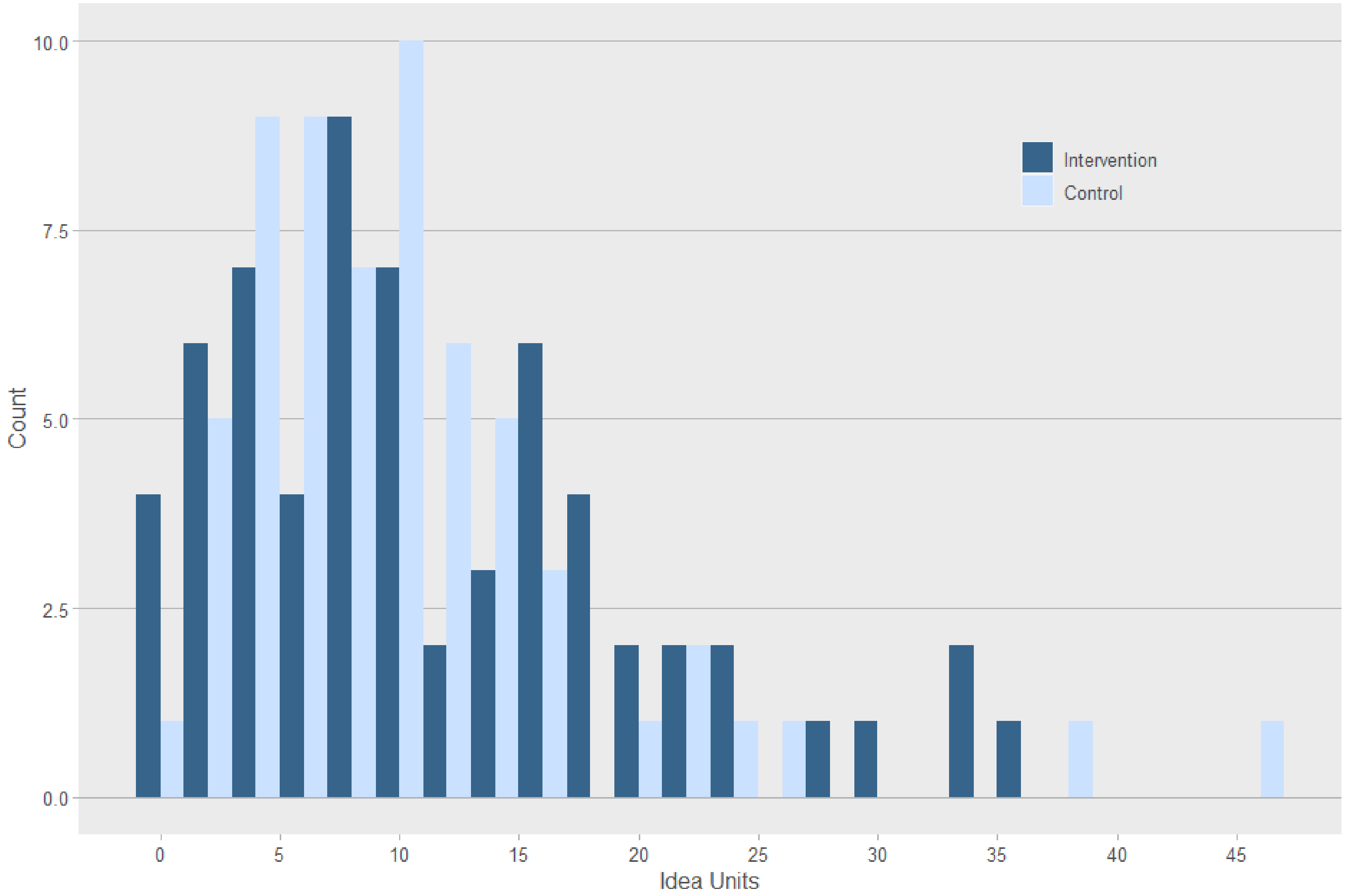

Supplement: Supplemental Digital Appendix 5. — Plots of student responses on study outcomes. [file pme-12-1-1017-s5.pdf]
